# Non-Radiation-Related Osteonecrosis of the Jaws in Dogs: 14 Cases (1996–2014)

**DOI:** 10.3389/fvets.2015.00007

**Published:** 2015-05-05

**Authors:** Santiago Peralta, Boaz Arzi, Ana Nemec, Milinda J. Lommer, Frank J. M. Verstraete

**Affiliations:** ^1^Department of Clinical Sciences, College of Veterinary Medicine, Cornell University, Ithaca, NY, USA; ^2^Department of Surgical and Radiological Sciences, School of Veterinary Medicine, University of California Davis, Davis, CA, USA; ^3^Small Animal Clinic, Veterinary Faculty, University of Ljubljana, Ljubljana, Slovenia; ^4^Aggie Animal Dental Center, Mill Valley, CA, USA

**Keywords:** osteonecrosis of the jaws, osteomyelitis, mandible, maxilla, dogs

## Abstract

Osteonecrosis of the jaws (ONJ) is an entity of major clinical impact characterized by chronically exposed necrotic mandibular or maxillary bone. Its clinicopathological characteristics and possible inciting or risk factors are well described in humans but only anecdotally reported in dogs. Treatment modalities and outcome vary depending on the inciting factors involved and the extent and severity of the lesions. The objectives of this study were to retrospectively describe the clinicopathological features of non-radiation-related ONJ in a series of 14 dogs, identify possible inciting or risk factors, and report on the surgical treatment and outcome. For all patients, the medical records were used to collect information regarding signalment, clinical signs, characteristics of the oral, jaw and dental lesions, diagnostic imaging findings, histopathological and microbiological analysis, treatment performed, and outcome. The data collected showed that non-radiation-related ONJ appears to be an infrequent clinical entity but of significant impact in dogs; that a history of systemic antibiotics and dental disease is common among affected dogs; that previous dental extractions are commonly associated with ONJ sites; that using a systematic diagnostic approach is essential for diagnosis; and that thorough surgical debridement combined with a course of oral antibiotics was effective in the described dogs affected by advanced non-radiation-related ONJ.

## Introduction

Osteonecrosis of the jaws (ONJ) is a clinical term used in human medicine, and refers to chronically exposed necrotic mandibular or maxillary bone (1–4). A condition that resembles this has been anecdotally reported in dogs (5–7). The definitive diagnosis of ONJ is based on historical, clinical, diagnostic imaging, and histopathological findings (3, 8). Apart from exposed necrotic bone, possible clinical signs of ONJ include pain, presence of a draining tract, swelling, and lymphadenopathy. Systemic signs may include fever, deep pain, lethargy, and anorexia (9, 10).

Although well documented in humans, the pathophysiology of ONJ remains unclear (2, 11, 12). Identification of inciting causes or risk factors for ONJ may provide useful therapeutic and prognostic information. The most frequently identified inciting or risk factors reported in humans are the administration of certain systemic medications (e.g., bisphosphonates, denosumab, steroids), osteomyelitis due to odontogenic infection, oral surgery (e.g., extractions, orthognathic surgery, etc.), and radiation therapy to the head and neck (1, 3, 8, 13, 14). Other possible inciting causes include neoplasia, direct chemical toxicity (e.g., formocresol), and systemic toxicity (e.g., exposure to industrial phosphorus and radium) (1, 3, 9). Underlying factors are usually identified based on historical, clinical, diagnostic imaging, and histopathological findings; cases in which an inciting cause cannot be established are regarded as idiopathic (8).

The clinical consequences of ONJ can be devastating, and aggressive medical and surgical intervention is often warranted (9, 14). The management strategies for ONJ vary depending on the underlying process or risk factors involved, as well as the extent and severity of the ONJ lesions. In general, the main therapeutic goals are to eliminate pain, control infection, and stop the progression (15). In cases of mild or relatively asymptomatic ONJ, conservative treatment may be indicated including a combination of pain medications, topical antiseptic solutions, and oral antibiotics (10, 15). In more severe cases, surgical debridement, sequestrectomy, or *en bloc* excision of the affected area is indicated (1, 3, 9, 14, 15).

The clinical characteristics of ONJ, as well as the diagnostic imaging and histological features, and possible inciting causes or risk factors, have not been systematically described in dogs. The aim of this case series was to describe the clinicopathological features of non-radiation-related ONJ in dogs, identify possible inciting or risk factors, and report on the surgical treatment and outcome.

## Materials and Methods

### Criteria for selection of cases

The medical records of dogs diagnosed with ONJ, examined at the Companion Animal Hospital at Cornell University, Ithaca, NY, USA; the William R. Pritchard Veterinary Teaching Hospital at the University of California – Davis, CA, USA; and the Aggie Animal Dental Center, Mill Valley, CA, USA; between 1996 and 2014, were evaluated. Dogs were included if they had no previous history of radiotherapy. Cases, where ONJ was determined to be of neoplastic origin, or if there was a history of electric burns in the oral cavity, were excluded. The diagnosis of ONJ was based on clinical presentation and diagnostic imaging findings, and histopathological analysis of representative tissues when available.

### Procedures

Information obtained from the medical records included breed; age; gender (and reproductive status); body weight; clinical signs; physical and oral examination findings; location and number of ONJ sites; systemic, oral, and dental status; diagnostic imaging findings from intraoral radiographs and computed tomographic (CT) images of the skull; complete blood count (CBC), serum biochemistry and urianalysis results; histopathological and microbiological analysis; treatment performed; and outcome. All histopathological specimens were routinely processed for histopathological analysis using H&E staining. The study group was not compared to the overall patient population, and no statistical analysis was performed to compare the different variables due to small sample size and limitations collecting data from multiple institutions.

## Results

### Patient population

A total of 14 dogs met the criteria for inclusion and the relevant signalment information is summarized in Table 1. The age of the patients ranged from 4 months to 13 years (mean = 8.2, median = 8). The sex distribution was six males and eight females. The reproductive status was one intact male and four intact females, representing a total of five intact animals; and five neutered males and four spayed females, representing a total of nine altered animals. The body weight of the animals ranged from 10 to 50 kg (mean = 21.1, median = 17.4).

**Table 1:** **Signalment information of the patient population**.

Dog #	Breed	Age (years)	Reproductive status	Body weight (kg)
1	Scottish Terrier	6	FS	10.4
2	Cocker Spaniel	11	FS	10.4
3	Scottish Terrier	4	M	10
4	German Shepherd	10	MC	26
5	Pit Bull	13	F	25.6
6	Cocker Spaniel/Corgi Mix	5	MC	17.6
7	English Springer Spaniel	11	FS	36
8	Airedale Terrier	8	F	26
9	Saint Bernard	8	F	50
10	Scottish Terrier	7	F	11.2
11	Labrador Retriever	11	FS	31
12	Scottish Terrier	5	MN	10
13	Wheaten Terrier	9	MN	17.2
14	Cocker Spaniel	7	MC	13.4

*F, female; FS, female spayed; M, male; MC, male castrate*.

### Historical and physical examination findings

Most dogs were presented because of non-resolving oral or dental-related clinical signs with a duration ranging from 0.3 to 36 months (mean = 5.7, median = 3) (Table 2). Recent previous dental extractions due to oral/dental disease were reported in 11 dogs; 10 of these dogs had at least 1 ONJ site that coincided with areas of recent dental extractions. Nine dogs were receiving or had recently received courses of oral antibiotics prescribed due to oral/dental problems, 7 of which had ONJ at sites of recent dental extractions. Other relevant concurrent medical conditions were reported in seven dogs but none were presumed to have a direct association with the chief complaint, or precluded the patients from general anesthesia, or any of the diagnostic or therapeutic procedures related to ONJ. The most common clinical signs recorded were halitosis in 14 dogs, mandibular lymphadenopathy in 11 dogs, oral pain in 9 dogs, local swelling in 3 dogs, inappetence or difficulty eating in 6 cases, lethargy in 3 dogs, drooling in 1 dog, and weight loss in 1 dog. Fever was not detected in any of the dogs.

**Table 2:** **Relevant historical data and physical examination findings at presentation**.

Dog #	Presenting complaint	Duration (months)	Reported medical conditions	Recent dental extractions	Recent antibiotic therapy	Relevant clinical signs at presentation
1	Osteonecrosis	4	Epilepsy, hypothyroidism	Yes	Yes	H, ML, I/DE, OP
2	Odontogenic infection	3	None reported	Yes	No	H, ML
3	Osteonecrosis	2	Ear disease, benign prostatic hyperplasia	Yes	Yes	H, ML, I/DE, OP
4	Osteonecrosis	2.5	None reported	Yes	Yes	H, ML, I/DE, OP, LS, L
5	Osteonecrosis	1	None reported	Yes	Yes	H, L
6	Osteonecrosis	5	Systemically healthy; historic hemilaminectomy	Yes	Yes	H, OP
7	Coughing	3	Blind, orthopedic disease	No	Yes	H, ML, OP
8	Osteonecrosis	1.5	First degree block, heart murmur	Yes	No	H, ML
9	Jaw mass	3	None reported	No	Yes	H, I/DE, OP, L
10	Oral mass	6	None reported	Yes	Yes	H, ML
11	Jaw mass	0.3	Prosthetic hips, sensitive to NSAIDs, hypothyroidism	No	No	H, ML, I/DE, OP, LS, PT
12	Severe halitosis, lethargy	12	None reported	Yes	Yes	H, OP, L
13	Severe halitosis and dental disease	36	None reported	Yes	No	H, ML, LS
14	Jaw mass	1	Mitral valve degeneration	Yes	No	H, ML, OP, LS

*H, Halitosis; ML, mandibular lymphadenopathy; OP, oral pain; I/DE, inappetence/difficulty eating; L, lethargy; LS, local swelling; PT, ptyalism*.

### Hematological findings

A CBC was performed in 12 dogs; results were unremarkable in 8 dogs. In the other dogs, the CBC abnormalities consisted of mild neutrophilia in one dog, mild neutrophilia and mild lymphopenia in one dog, mild leukopenia and mild non-regenerative anemia in one dog, and mild monocytosis in one dog. A complete serum biochemistry panel was performed in 12 dogs; results were unremarkable in 5 dogs. In the other seven dogs, abnormalities included a mild to moderate hyperglobulinemia in four dogs, mild to moderate elevation of ALP in four dogs, mild elevation of ALT in one dog, mild hypoalbuminemia in one dog, and mildly elevated BUN in one dog. A urinalysis was performed in six dogs; results were unremarkable in three dogs. In the other dogs, the urinalysis abnormalities detected consisted of isosthenuria in two dogs, and bacteriuria in one dog.

### Intraoral clinical findings

**Figure 1:**
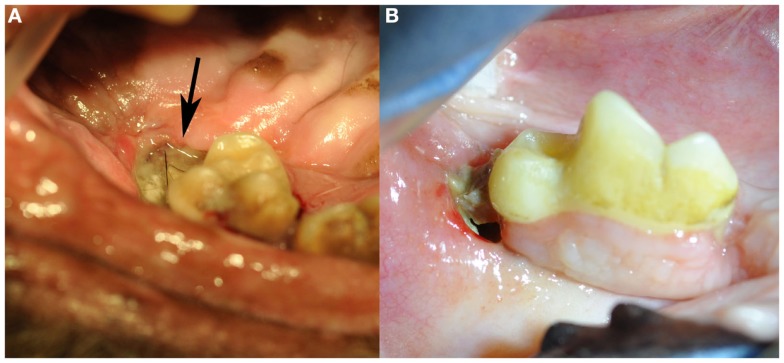
**Clinical images of non-radiation-related ONJ in dogs: (A) an ONJ site (case 7) located in the caudal left maxilla (black arrow)**. Note the missing second molar tooth, and the plaque and debris covering the exposed necrotic bone. **(B)** An ONJ site (case 14) located in the body of the right mandible. (Reprinted with permission from the American Veterinary Medical Association, article in press)

All dogs received a comprehensive oral and dental examination and full-mouth intraoral radiographs. A total of 22 ONJ sites (Table 3) characterized by intraoral areas of exposed necrotic bone were identified among the 14 dogs (range = 1–4; median = 1) (Figure 1). All ONJ sites involved alveolar bone regardless of the presence or absence of teeth, but often extended into the corresponding jawbone proper. The exposed bone was usually covered by plaque, debris (e.g., hair, food, etc.), and variable amounts of granulation tissue. Of the 22 ONJ sites, 13 (59.1%) were located on the maxilla and or incisive bone, and 9 (40.9%) on the mandible. Based on the medical records and intraoral radiographs available, 14 ONJ sites (63.3%) occurred in areas of recently extracted teeth.

**Table 3:** **Osteonecrosis sites and diagnostic tests performed in each animal**.

Dog #	ONJ sites	FMR	CBC	Chem	UA	CT	Histo	Cult
1	Caudal left mandible and maxilla	X						
2	LmaxP1–2 and LmaxP3 areas	X	X	X			X	
3	L+RmandP3–M1 areas	X	X	X	X	X		X
4	LmaxP4–M1 area	X	X	X	X			
5	RmaxP4 and LmaxM1 areas	X	X	X	X			
6	L+RmaxI1 areas	X	X	X			X	
7	LmaxP4–M2 area	X	X	X	X	X	X	X
8	LmaxC–M1 area	X	X	X		X	X	X
9	LmaxM1–M2 and LmandM2–3 areas	X	X	X	X			X
10	All quadrants	X	X	X	X			
11	LmandM1 area	X	X	X			X	
12	LmaxM1–M2 area	X					X	
13	LmandM1–M2 area	X	X	X	X			
14	RmandM1–M3 area	X	X	X		X	X	

*FMR, full-mouth radiographs; CBC, complete blood count; Chem, serum biochemistry; UA, urianalysis; CT, computed tomography; Histo, histopathology, Cult, bacterial culture*.

### Diagnostic imaging findings

**Figure 2:**
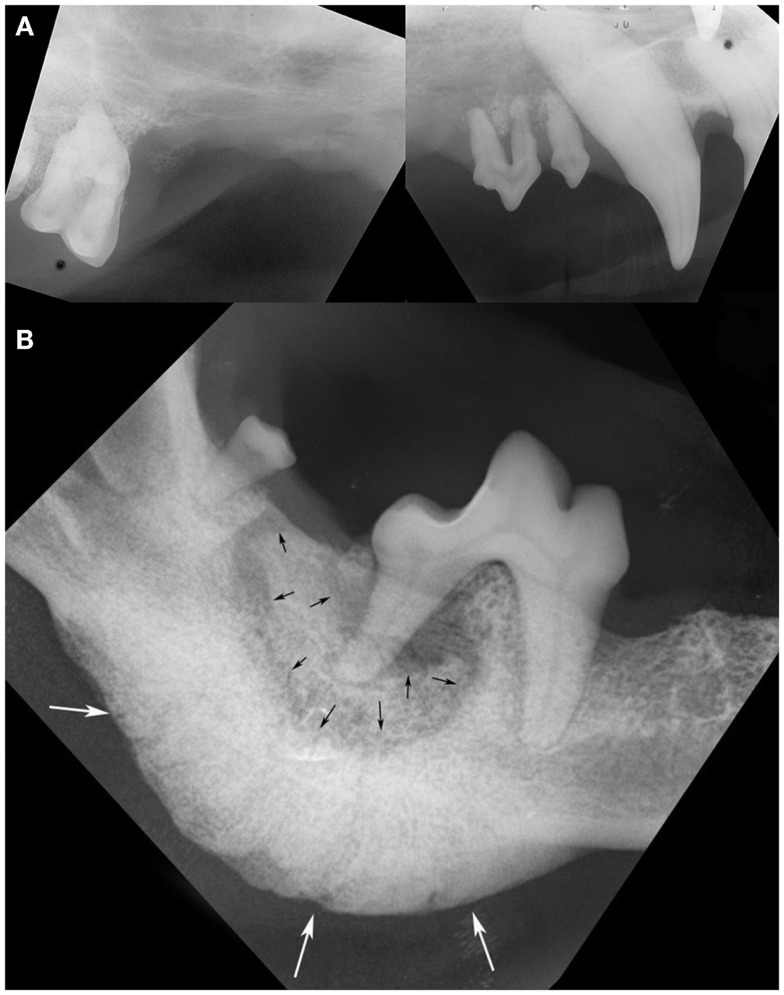
**Intraoral radiographs of ONJ sites: (A) a pattern of geographic bone loss extending from the right maxillary canine tooth to the right maxillary first molar tooth is observed (case 8)**. Note the loss of attachment and external inflammatory root resorption around the canine and premolar teeth. **(B)** A sequestrum (black arrows) surrounding the distal root of the right first mandibular molar tooth, and an associated severe solid periosteal reaction located on the ventral border of the mandible (white arrows) are evident in the same patient shown in Figure 1B (case 14). (Reprinted with permission from the American Veterinary Medical Association, article in press)

The most common intraoral radiographic findings at ONJ sites included areas of bone loss, and/or sequestrum formation, and/or a periosteal reaction (Figure 2). Radiographically, the areas of ONJ originated from areas where a tooth with pathological changes was present and/or from vacated alveoli where extractions had been performed recently. Although ONJ lesions were usually focal, they often extended beyond the clinically visible sites, and could involve adjacent dentate or edentulous areas, or extend deeper into the jawbone proper. One maxillary ONJ lesion was diffuse, extending caudally and dorsally beyond normal intraoral radiographic reach. When visible, the size of bone sequestra varied, and was or was not in direct association with a tooth with pathological changes. The areas of bone loss were usually diagnosed as having a moth-eaten or permeative pattern (16). The periosteal reaction observed was typically of the solid type (16, 17), and usually occurred around and/or was more severe in areas of bone sequestration. The diagnostic information obtained with intraoral radiographs from ONJ sites affecting the mandibles correlated well with CT imaging findings and/or surgical exploration of the lesions. In contrast, intraoral radiographs often significantly underestimated the nature, severity, and extent of lesions affecting the maxilla, and correlated poorly with CT imaging findings and/or surgical exploration of the lesion. Pathological changes affecting teeth at the lesion site or in the immediate vicinity were common and included varying degrees of alveolar bone loss, periapical lucencies, and areas of external inflammatory root resorption (18).

**Figure 3:**
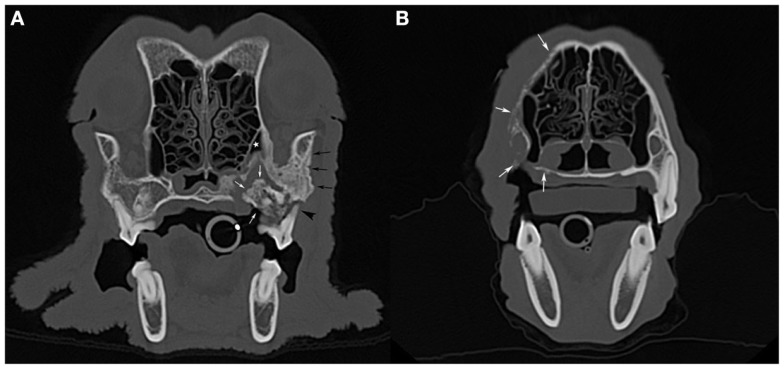
**Computed tomographic images: (A) an axial slice image of the skull of the dog imaged in Figure 1A (case 7), with a large sequestrum at the left caudal maxilla (white arrows) that extends into the maxillary recess (white star)**. Note the associated periosteal reaction (black arrows) and total loss of attachment at one of the buccal roots of the left maxillary first molar tooth (black arrowhead). **(B)** An axial slice image of a dog with bone lysis of the right maxilla (white arrows) that extends from the alveolar bone and palatal process to the nasal bone dorsally and toward the midline along the hard palate. The extent of the lesions in this case (case 8) was not suspected clinically or radiographically (see Figure 2A).

A skull CT scan was performed in four dogs. In all cases, the main CT finding consisted of bone loss of a moth-eaten appearance that extended beyond clinically and radiographically detectable ONJ sites. A solid periosteal reaction around affected areas was present in three cases. Sequestrum formation at the ONJ site was detected in two dogs, including one sequestrum located in the caudal maxilla that extended into the maxillary recess, and which had not been detected radiographically (Figure 3A). In one case of a maxillary ONJ in which intraoral radiographs did not reveal the extent of the lesion, the CT images showed that there was severe bone lysis that involved the entire left maxillary bone, extending dorsally toward the nasal bones, rostrally to the level of the canine tooth, and caudally into the rostral third of the zygomatic arch (Figure 3B). The soft tissues surrounding ONJ sites appeared mildly thickened and were moderately contrast-enhancing. Marked to severe regional lymphadenopathy was observed on CT images in all four cases.

### Concurrent dental status

Combined, clinical and radiographic findings revealed concurrent oral and/or dental disease in all dogs. The findings included horizontal and/or vertical alveolar bone loss, moderate gingivitis, furcation involvement or exposure, gingival recession, and root exposure, consistent with periodontitis. The extent and severity of periodontitis varied between individuals and ranged between mild localized to severe generalized; the teeth affected were distant to and/or directly associated with ONJ sites. Severe diffuse chronic stomatitis was present in the four Scottish terriers included in the study.

### Histopathological findings

**Figure 4:**
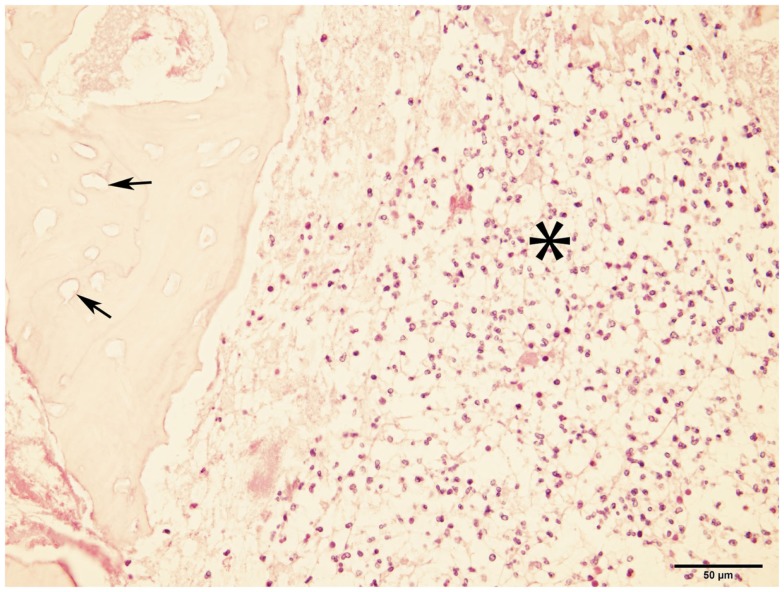
**Micrograph of bone from ONJ site**. The image shows necrotic bone characterized by empty lacunae (arrows), and surrounded by a bone marrow space infiltrated with inflammatory cells (asterisk). These findings are consistent with osteomyelitis (H&E, ×40 magnification).

Bone samples from ONJ sites were obtained from seven dogs. Histopathological findings included the presence of necrotic bone with empty lacunae, and marrow space containing variable and often abundant amounts of neutrophils, lymphocytes, macrophages, plasma cells, and osteoclasts (Figure 4), consistent with chronic osteomyelitis. Bacterial colonization of the tissue was observed in all but one case; no fungal elements were detected in any of the cases.

### Microbiological results

Microbiological samples from the ONJ site were obtained from five dogs. Cultures were positive in four dogs and were all polymicrobial in nature. Aerobic bacteria were isolated from three dogs; isolated organisms included *Streptococcus canis*, *Enterococcus faecalis*, *Actinomyces* spp., *Escherichia coli*, and *Pseudomonas aeruginosa*. Anaerobic bacteria were isolated from three dogs; isolated organisms included *Fusobacterium nucleatum*, *Peptostreptococcus* spp., *Bacteroides* spp., and *Porphyromonas* spp.

### Treatment implemented and outcome

**Figure 5:**
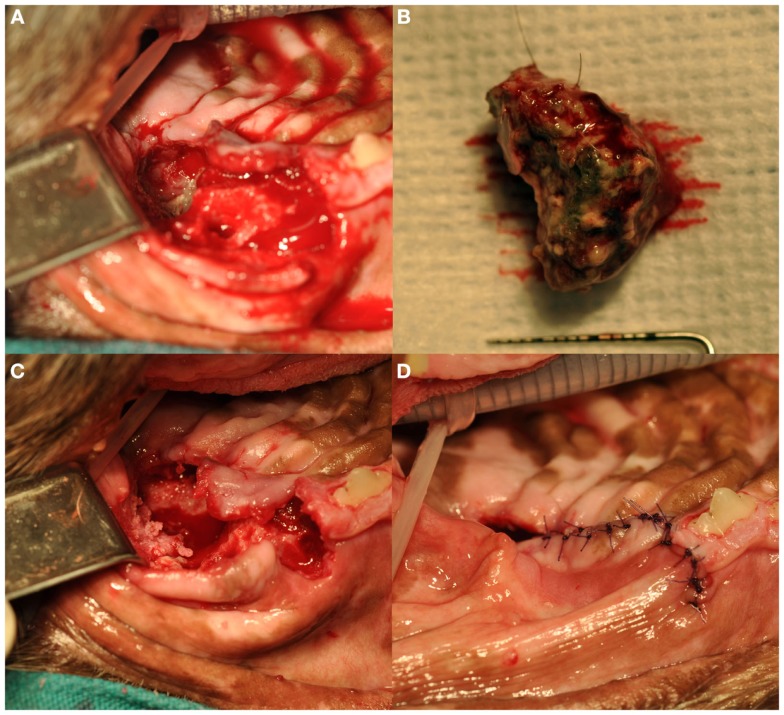
**Surgical debridement and sequestrectomy at the left caudal maxillary ONJ site of the dog shown in Figures 1A and 3A (case 7): (A) the area of ONJ after a full-thickness mucogingival-periosteal flap had been raised, and the fourth premolar and first molar teeth had been extracted**. **(B)** A large sequestrum that was removed from the area. **(C)** The surgical site after sequestrectomy and debridement. Note the underlying healthy bleeding bone. **(D)** The flap during closure.

Treatment of ONJ was surgical in all dogs (Figure 5). Immediately prior to surgical treatment, all dogs received an intravenous dose of 20 mg/kg of ampicillin, followed by complete periodontal treatment, regional nerve blocks as indicated by the surgical plan, and dental extractions following standard technique (19), as indicated by clinical and radiographic findings. In 13 dogs, surgery consisted of exposing the ONJ sites by elevating full-thickness mucogingival-periosteal flaps of appropriate size based on clinical and diagnostic imaging findings. Once exposed, all necrotic bone and associated granulation tissue were removed, and sequestrectomy was performed when indicated. Bone debridement was performed using a round diamond bur on an air-driven high-speed handpiece or a bone cutting tip on a piezoelectric surgical handpiece, until bleeding from the underlying bone occurred. The site was then copiously rinsed with sterile saline, and closed using absorbable suture material. All teeth involved with the ONJ process, as determined by clinical and diagnostic imaging findings, were extracted using standard extraction technique. The surgical treatment in one dog consisted of *en bloc* excision of the ONJ site via incisivectomy.

A course of oral post-operative antibiotic therapy was prescribed in all cases. The antibiotics used included amoxicillin/clavulanic acid, metronidazole, or clindamycin. The duration of antibiotic therapy ranged between 2 weeks and 2 months. Additional treatments prescribed included post-operative analgesics and a daily oral rinse with 0.05–0.12% chlorhexidine gluconate solution. Follow-up was available for 12 dogs and ranged between 1 and 15 months (mean = 3 months, median = 1.25 months). Healing of the surgical site was documented in 12 dogs that received follow-up. Chronic stomatitis persisted and periodontal disease progressed in the four Scottish terriers; one of these four dogs developed ONJ at a different site 10 months later and an additional surgical intervention was performed, but the dog was eventually lost to follow-up.

## Discussion

This case series describes the clinicopathological features and possible inciting factors of non-radiation-related ONJ in dogs, and reports on the surgical treatment and outcome. The clinical manifestations of ONJ observed in the dogs reported here were similar to those described previously in humans and dogs (5–7), and are illustrative of the potential impact on the health status and quality of life of affected individuals. Even though the patient population included was not compared to the overall hospital populations, the prevalence of non-radiation-related ONJ in dogs appeared low: 14 dogs in a period of 18 years were presented to 3 different referral institutions. Additionally, no apparent association between ONJ and gender, or body size was observed in the sample included.

Noteworthy is that the breeds Scottish terrier (4 of 14 dogs) and cocker spaniel/cocker mix dogs (3 of 14 dogs) accounted for half of the animals within the study group. Consistent with a previous report (20), the clinical presentation of ONJ in the four Scottish terriers included concurrent non-resolving chronic stomatitis and progressive periodontitis. A possible breed predisposition to ONJ in Scottish terriers, and in cocker spaniels as previously suggested (6) is possible. The four Scottish terriers reported here were all young or middle-aged adults, younger than the average age of the study group. Of the two similar cases previously reported (20), one was also a young adult (4-year-old). Whether these findings support specific breed predispositions, or reflect a different clinical entity or syndrome requires further investigation.

Consistent with some of the risk factors identified in humans (8, 13, 21), all dogs in this study were diagnosed with some form of dental or oral disease, and all but one had a history of recent dental extractions (with more than half of the ONJ sites associated with an area of recent extractions) and/or had recently received courses of oral antibiotics. Although it is plausible that the surgical manipulation during dental extractions and/or antibiotics predisposed the dogs to ONJ, their exact pathogenic role cannot be established based on this study. Moreover, it is not possible to retrospectively determine if ONJ developed as a result of the underlying dental disease that warranted extractions and antibiotic therapy, or if the two latter caused the lesions. Conversely, none of the dogs had a history of concurrent or previous administration of bisphosphonates. An association between ONJ and bisphosphonates in humans is well documented (18, 22, 23). In dogs, bisphosphonate-related ONJ has been studied under experimental conditions (23–26), but no naturally occurring cases have been documented.

Despite the severity of some of the lesions detected and the associated local and systemic clinical signs, no cases of fever were detected and no obvious patterns of hematological and serum biochemistry abnormalities were observed different from mild changes attributable to chronic inflammation. Conversely, the results of other diagnostic tests performed were crucial for establishing a diagnosis and implementing a surgical plan. In particular, radiographic and CT findings allowed sufficient diagnostic information to execute a surgical treatment plan, and were consistent with those previously reported in humans (27, 28). Radiologically, the lesions consisted of areas of bone lysis, sequestrum formation, and periosteal reactions of varying severity, all of which constitute typical features of ONJ and chronic osteomyelitis (27–29).

In contrast with mandibular intraoral radiographs, maxillary intraoral radiographs offered inconsistent and unreliable diagnostic information regarding ONJ lesions. Such difference is likely associated with the implicit limitations of the technique used to image the maxilla (i.e., bisecting angle technique) compared to the technique used to image the body of the mandible (i.e., parallel technique) (30); and to the amount of superimposed structures on maxillary radiographs (e.g., nasal cavity, multi-rooted teeth, sinuses, sutures, etc.) compared to radiographs of the mandibular body. In contrast, as has been reported (27, 28), CT was found to be a valuable and consistent diagnostic modality that also allowed a precise surgical plan to be made.

The histopathological findings revealed chronic osteomyelitis in all cases analyzed. In humans, chronic osteomyelitis of the jaws is usually secondary to odontogenic infections, and often results in sequestrum formation and ONJ (1, 4). Although the historical and clinicopathological information collected from the cases described here is compatible with a diagnosis of ONJ secondary to osteomyelitis of odontogenic origin, as previously mentioned, the exact disease mechanisms involved and potential risk factors could not be ascertained.

Bacteria are believed to play a major role in the pathophysiology of ONJ including ONJ secondary to osteomyelitis, induced by bisphosphonates, and osteoradionecrosis (8, 22, 31, 32). As was the case in the majority of the bacterial results described in this report, the presence of bacteria at ONJ sites is usually polymicrobial in nature, and may reflect the flora of the oral cavity (10, 28). Regardless of their pathophysiological role, in contrast with all other diagnostic tests performed, the bacterial culture results reported here had a minimal impact on treatment planning or outcome. Additionally, it is likely that the intravenous perioperative antibiotic dose administered to all patients, and the historical oral courses of oral antibiotics in nine of the affected dogs, altered the original microbial population at ONJ sites. Until the role of specific bacterial species or groups of cohabiting bacteria is established in regards to the pathogenesis of ONJ in dogs, the clinical, therapeutic, and prognostic information obtained from bacterial cultures will be limited to those aspects related to susceptibility and proper antibiotic selection for individual patients.

The treatment of severe or advanced ONJ is typically surgical regardless of possible inciting factors, and is often followed by a course of oral antibiotics. Surgical intervention consists of the removal of necrotic or infected bone or associated tissues until grossly healthy, bleeding tissues are encountered (5, 7, 9, 18, 27). Based on the follow-up information, the results of this study show that the surgical debridement of ONJ sites combined with a course of oral antibiotics is a highly effective treatment modality in dogs.

We conclude that non-radiation-related ONJ is an infrequent clinical entity but of significant impact in dogs. Additionally, dental disease and/or a history of previous dental extractions and oral antibiotics are common among affected dogs, and these may represent risk factors or play a causative role. This study also highlights the importance of following a systematic diagnostic approach in cases of ONJ, including the use of diagnostic imaging modalities and histopathological analysis. Finally, we conclude that aggressive surgical debridement combined with a course of oral antibiotics was effective in the described dogs affected by advanced non-radiation-related ONJ.

## Conflict of Interest Statement

The authors declare that the research was conducted in the absence of any commercial or financial relationships that could be construed as a potential conflict of interest.
